# *Vernonia amygdalina* simultaneously suppresses gluconeogenesis and potentiates glucose oxidation via the pentose phosphate pathway in streptozotocin-induced diabetic rats

**DOI:** 10.1186/1472-6882-14-426

**Published:** 2014-10-30

**Authors:** Item Justin Atangwho, Khoo Boon Yin, Muhammad Ihtisham Umar, Mariam Ahmad, Mohd Zaini Asmawi

**Affiliations:** School of Pharmaceutical Sciences, Universiti Sains Malaysia, Minden, 11800 Penang Malaysia; Institute for Research in Molecular Medicine (INFORMM), Universiti Sains Malaysia, Minden, 11800 Penang Malaysia; Department of Biochemistry, College of Medical Sciences, University of Calabar, P.M.B. 1115, Calabar, Nigeria

**Keywords:** *Vernonia amygdalina*, Gene expression, Gluconeogenesis, Pentose phosphate patheway, Diabetes mellitus, Mechanism of anti-diabetic action

## Abstract

**Background:**

This study evaluated the impact of *Vernonia amygdalina* (VA) on the transcription of key enzymes involved in cellular modulation of glucose in streptozotocin-induced diabetic rats in a bid to understand the possible anti-diabetic mechanism of VA.

**Methods:**

The chloroform fraction of VA (200 mg/kg and 400 mg/kg body weight) was administered to SDRs for 7 and 14 days. Thereafter, the expression (transcription) of key carbohydrate regulatory genes was evaluated in selected tissues - adipose, muscle and liver. Also, the body weight and blood glucose changes were monitored.

**Results:**

A 14-day administration of 200 mg and 400 mg of the extract and metformin (500 mg/kg) showed a striking decrease (*P* <0.05) in the expression of the gluconeogenic enzymes - fructose 1,6-bisphosphatase, phosphoenol pyruvate carboxykinase and glucose 6-phosphatase in the liver and muscle compared to the diabetic control. These genes were highly expressed in tissues of untreated diabetic rats (*P* <0.05) indicating severe gluconeogenesis. Furthermore, the extract as well as metformin significantly increased glucose oxidation via the pentose phosphate pathway (PPP) i.e. increased expression of the glucose 6-phosphate dehydrogenase (G6PDH) gene (*P* <0.05) in the liver. Conversely, the expression of the G6PDH in the muscle and adipose tissues significantly decreased (*P* <0.05), suggesting enhanced utilization of NADPH and ribose in the clearance of reactive oxygen species and for expression of other relevant genes respectively. Also, transcription of the cell proliferation regulatory enzyme, phosphatidylinositol 3-kinase increased in the liver, but decreased in the muscle and adipose tissues (*P* <0.05) upon treatment with the extract or metformin, implying that the liver responded to the VA and metformin treatments more than other organs. The extract administration also caused a decrease in the expression of key enzymes of glycolysis namely hexokinase and phosphofructokinase, suggestive of a glucose sparing for ribose and NADPH production in PPP.

**Conclusion:**

Overall, data obtained in this study suggest that VA exerts little or no effect on glycolysis; rather, it may achieve its anti-diabetic action by a simultaneous suppression of gluconeogenesis and potentiation of glucose oxidation via PPP pathway, almost exclusively in the liver.

## Background

Diabetes mellitus is described as a disease syndrome with a collection of metabolic disorders characterized by chronic hyperglycemia which results from defects in insulin secretion, insulin action, or both [1, 2]. The chronic metabolic disorders include, but are not restricted to alterations in the metabolism of major energy molecules - carbohydrates, fats, and proteins [3] that usually culminate in complications of diabetes. This alteration in the metabolism of the energy molecules derives largely from changes in the activities of defining proteins or enzymes of glucose smetabolism or transport in target tissues – liver, muscle and adipose. Consequently these key proteins and/or enzymes constitute important checkpoints in the endogenous and exogenous glucose homeostasis; hence can be exploited in the mechanistic study of both conventional and potential anti-diabetic drugs.

Several conventional anti-diabetic drugs are known to exert their therapeutic actions via modulation of these molecular targets. For instance, the thiazolidinediones (TZDs) exert their anti-diabetic action by binding to the peroxisome proliferator-activated receptor-gamma (PPAR-γ), a nuclear receptor that regulates the transcription of specific genes involved in glucose and lipid metabolisms and energy balance (e.g. lipoprotein lipase, fatty acid transporter protein, adipocyte fatty acid binding protein, fatty acyl-CoA synthase, malic enzyme, glucokinase and the GLUT4 glucose transporter genes), in a way that improves insulin sensitivity in adipose, muscle and the liver tissues [4, 5]. On their part, the biguanides such as metformin function as anti-diabetic drugs by decreasing hepatic glucose production, mainly by inhibiting hepatic gluconeogenesis or reduction in hepatic uptake of gluconeogenic substrates and the stimulation of glucose uptake in muscle [6]. These effects are achieved by adenosine monophosphate kinase (AMPK) - mediated transcriptional regulation of genes involved in gluconeogenesis in the liver and those encoding glucose transporters in the muscle, such as peroxisome proliferator-activated receptor-γ coactivator 1α and glucose transporter type 4, respectively, consequently enhancing tissue insulin sensitivity and lowering of fasting blood glucose [7]. By a similar token, mechanistic studies are usually designed to evaluate the anti-diabetic mechanism of novel drugs including extracts by assaying the expression of genes of key enzymes or proteins involved in glucose transport and biotransformation, fat modulation and cell proliferation.

The traditional claim of the use of *Vernonia amygdalina* Del. (VA) leaves in treatment of diabetes has long been pharmacologically validated as possessing the potential to lower blood glucose level in experimental diabetic models [8–10]. In furtherance we demonstrated in a recent study that the sequential chloroform extract rather than the methanol or water extracts of the leaves of VA is most potent in exerting anti-diabetic activity; and that the dominant fatty and phytanic acids in this fraction were most probably responsible for the observed effect [11]. Although several bioactive compounds have been isolated from VA including Vernodalin, Vernomygdin, Vernoniosides (A1, A2, A3, A4, B1, B2, B3, D & E), Vernodalol and Epivernodalol [12], none of these isolated compounds is shown to be responsible for the anti-diabetic properties of VA. Moreover, very few studies have considered the mechanism of anti-diabetic action of VA, beyond the scientific validation of the traditional claim. In some previous studies, administration of VA extract was shown to cause a regeneration of the β-cells of the pancreas [9, 10, 13], thereby contributing in part to its anti-diabetic effect. These reports on mechanism are scanty, since the derangements soon after outset go beyond the β-cell sequestration, such that regeneration of β-cell alone may not entirely address the complications. It was therefore necessary to study the impact of VA on the metabolism of carbohydrate, the biomolecule whose metabolism is s grossly affected in diabetic condition.

Consequently, the purpose of the present study was to evaluate changes in mRNA expression of key glucose modulatory enzymes induced by the active chloroform extract of VA leaves on the liver, muscle and adipose tissues, in order to achieve a deeper understanding of the molecular changes associated with the reported anti-diabetic activity.

## Methods

### Preparation of plant extract and fraction

The leaves of *Vernonia amygdalina* Del. were collected from a reserve at No. 16, Jalan Bukit Gambir, Penang, Malaysia. The leaves were identified and a voucher specimen deposited in the herbarium unit at the School of Industrial Biotechnology, Universiti Sains Malaysia (voucher number: 11341) for future reference. The extraction was performed according to the procedure described previously [11]. Briefly, about 700 g of oven-dried and powdered leaves were sequentially extracted by maceration in petroleum ether and chloroform at 40°C. The solvents were replenished daily for 3 days each, and on the 3rd day, the combined extracts were separately filtered with Whatman No. 1 filter paper and concentrated *in vacuo* at 40°C. The concentrated extracts were freeze-dried to obtain the petroleum ether extract and chloroform extracts, respectively. The chloroform extract, the most potent glucose-lowering extract as indicated from previous studies was stored in the freezer (-4°C) for use in the animal experiments.

### Animals

Sixty (60) male Sprague Dawley rats (200–250 g) were obtained from the Animal Research and Service Centre (ARSC), Universiti Sains Malaysia (USM). The animals were acclimatized to conditions in the animal transit room (23 ± 5°C, 50 ± 10% relative humidity, and 12 h dark/light cycle) at the School of Pharmaceutical Sciences, USM, where the animal experiments were carried out. Food and water were provided *ad libitum* but occasionally withdrawn as appropriate for each stage of the procedure. The animal procedures were approved by the Animal Ethics Committee, Universiti Sains Malaysia [Approval number: USM/Animal Ethics Approval/2012/ (76) (373)].

Diabetes was induced by intraperitoneal injection of 55 mg/kg b.w. of streptozotocin (Sigma, St Louis, MO, USA) to overnight fasted rats. After 72 h of STZ injection, fasting blood glucose (FBG) was measured using the Accu-check Advantage II Glucose meter (Roche Diagnostics Co., USA) and rats with FBG (mmol/L) ≥15 ≤ 25 were considered diabetic and used in the experiment.

### Assessment of anti-hyperglycemic activity

The anti-hyperglycaemic activity of the extract was assessed in diabetic rats by measurement of changes in FBG during and at the end of the 14-day study period. The rats were divided into 5 groups (n =12). Groups 1 and 2 served as normal and diabetic controls, respectively and both received equivalent volume of 5% Tween 80 (p.o.). Groups 3–5 received 500 mg/kg of metformin and 200 mg and 400 mg/kg of the chloroform extract (all reconstituted in 5% Tween 80), respectively. The treatments were administered in half doses (i.e., 250, 100 and 200 mg/kg, respectively) twice per day (morning and evening) during the 14-day period, to as much as possible provide for a continuous residual amount of the drug in the animals’ system, hence improved efficacy. The 14-day administration of extract and distilled water which commenced soon after the confirmation of diabetic status was done using oral gavages. Fasting blood glucose (FBG) and body weight were measured 3 days prior to streptozotocin injection and on days 0 (72 h after streptozotocin injection), 3, 7, 11 and 14. Six rats each from the 5 groups selected on days 7 and 14 (the end of the study), were sacrificed after euthanasia, and the liver, soleus muscle and epididymal adipose tissues surgically removed for gene expression analysis. The tissues were immediately rinsed in ice-cold saline (0.9% NaCl), put in falcon tubes containing TRI reagent and stored at -80°C until used for RNA extraction.

### Total RNA extraction and integrity/purity assessment

Total RNA was extracted from the tissues using TRI Reagent, combined phenol and guanidine thiocyanate in a mono-phase solution obtained from Molecular Research Center, Inc. 5645 Montgomery Road, Cincinnati, Ohio 45212, USA following the manufacturer’s protocol. Briefly, 50–100 mg of the tissue homogenized in 1 ml of TRI Reagent was phase separated into the aqueous, interphase and organic phases by addition of Bromochloropropane (BCP). After centrifugation at 12,000 g for 15 min at 4°C, the uppermost aqueous phase was aspirated and RNA precipitated from the aqueous solution using isopropanol. The precipitated RNA was washed with 75% ethanol, solubilized with DEPC water and stored at -80°C for further use after integrity and purity assessment. The integrity of the extracted RNA was measured by agarose gel electrophoresis: A molten 1% (w/v) solution of agarose in 0.5% Tris-borate-EDTA (TBE) buffer, stained with 3 μL Ethidium bromide was prepared and gently poured onto a cast positioned with a small comb (to define the sample application wells) and allowed to set at room temperature for 20–30 min. The gel was mounted in the electrophoresis tank and 0.5% TBE buffer added to cover the gel. The extracted RNA sample mixed with loading dye (GelRed™) in a 1: 3–5 ratios was gently applied onto the wells alongside the control, and the electrophoresis run at 95 V and 400 mA for 35 min. The gels were examined under a UV transilluminator, and samples that displayed the discrete 28S (approx. 5 kb) and 18S (approx. 2 kb) ribosomal RNA bands possessed the required integrity. The purity and yield/concentration of the extracted RNA was quantified using an Eppendorf nano spectrophotometer (Thermo Fisher Scientific NanoDrop 8000 spectrophotometer 3411 Silverside Road, Bancroft Building Wilmington, DE 19810 USA) at 260 and 280 nm. Samples with absorbance ratio A260/280 ≥ 1.8 were considered pure and used for subsequent experiments.

### First strand cDNA synthesis or reverse transcription of RNA to cDNA

The Revert Aid First Strand cDNA Synthesis Kit #k1621, #1622, Lot 00103407 (Thermo Scientific, Wilmington, DE 19810 USA) was used for the cDNA synthesis and the manufacturer’s prescribed procedure followed. However, prior to cDNA synthesis, the volumes of the extracted RNA was adjusted by dilution with nuclease free water to provide an equimolar concentration of the template RNA (25 ng/μL) in all samples. Where not used immediately the reverse transcription product was stored at -80°C until used. The success of the reverse transcription reaction was validated with conventional PCR amplification using a house keeping gene – GAPDH (Glyceraldehyde-3-phosphate dehydrogenase).

### Amplification of cDNA by conventional PCR

The conventional amplification of the synthesized cDNA was performed using high capacity Master Mix (0.05 U/μL *Taq* DNA polymerase, reaction buffer, 4 mM MgCl_2_, and 0.4 mM of each dNTP - dATP, dCTP, dGTP and dTTP) obtained from Thermo Scientific, Wilmington, DE 19810 USA. The total volume (25 μL) for one conventional PCR reaction consisted of the following: 12.5 μL Master Mix, 1 μL each of forward and reverse primers, 5 μL of template cDNA and 5.5 μL of nuclease free water. The MJ Research PTC-200 Peltier Thermal Cycler (590 Lincoln Street Waltham, Massachusetts 02451, USA) was used for the amplification with the cycle parameters shown in Table 1.

**Table 1:** **Outline of thermal cycling conditions**

Step	Temperature (°C)	Time	Number of cycles
Initial denaturation	94	10 min	1
Denaturation	94	20 s	40
Annealing	55	20 s	
Extension	72	30 s	
Final extension	72	10 min	1
Cool down	4	-	-

The primers used in this study were synthesized by Integrated DNA Technologies, San Diego, USA and their particulars are shown in Table 2. The PCR products mixed with GelRed™ dye were electrophoresed in electrophoresis tank using 0.5% TBE buffer and 2% agarose gel at 80 V run for 90 min. Gels were visualized under UV light and subjected to densitometry analysis (FlouChem FC2 software) for numerical transposition and normalization where GAPDH, the loading control was used as a reference gene. In each assay, a no-DNA control was incorporated to check for possible contamination.

**Table 2:** **Primer sequence and particulars**

Gene	Primer	Sequence	Amplicon	Tm (°C)
GK	Forward	5′-CATATGTGCTCCGCAGGACTA-3′	105 bp	61.7
	Reverse	5′-CTTGTACACGGAGCCATCCA-3′		
HK	Forward	5′-ACCCACGAAACAACACCATCA-3′	69 bp	79
	Reverse	5′-GACGTACAACAATGGCTCACTAAAG-3′		
PFK-1 (L)	Forward	5′-TTACCGATCACCCTCGTTCCT-3′	80 bp	84
	Reverse	5′-TTCCCCTTAGTGCTGGGATCT-3′		
PFK-1 (M)	Forward	5′-GTGGACACTGATATGACCATTGG-3′	67 bp	82
	Reverse	5′-ATGGCGTCCACGATCTCTACA-3′		
G6Pase	Forward	5′-GGATCTACCTTGCGGCTCACT-3′	132 bp	62.7
	Reverse	5′-TGTAGATGCCCCGGATGTG-3′		
F16BP	Forward	5′-CGTCCTATGCTACCTGTGTCCTT-3′	73 bp	81
	Reverse	5′-CCCCTCTTCTCGGGCTCTATTAT-3′		
PEPCK	Forward	5′-GCAACTTCTCTCGGCTCGTT-3′	99 bp	62.7
	Reverse	5′-TGGCAGTTCTACTGGGCTACAC-3′		
G6PDH	Forward	5′-GATGGCCTTCTACCCGAAGAC-3′	72 bp	82
	Reverse	5′-GCGGATGTCATCCACTGTGA-3′		
P13K	Forward	5′-CCTGAAAGAGCTGGTGCTACATT-3′	71 bp	81
	Reverse	5′-GTGTGACATTGAGGGAGTCATTG-3′		

### Statistical analysis

The data were analyzed by the analysis of variance (ANOVA) followed by the Dunnet test for post hoc analysis, using the SPSS professional software, version 17.0. Differences were considered significant at *P* <0.05.

## Results

### Body weight and blood glucose

**Figure 1:**
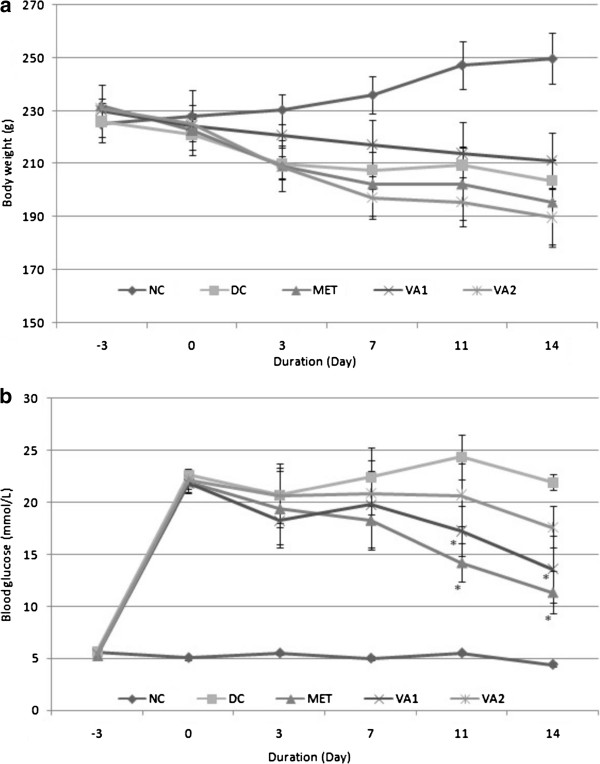
**Body weight (a) and blood glucose concentration (b) of streptozotocin-induced diabetic rats treated with chloroform fraction of** ***Vernonia amydalina*** **leaves.** Values are expressed as the mean ± SEM; n = 6; * = *P* <0.05 vs DC.

The body weight and blood glucose changes in response to a 14-day administration of the active chloroform extract of *Vernonia amygdalina* Del. leaves to STZ- induced diabetic rats are shown in Figure 1. The animals lost 5 – 10 g (2-4%) body weight and a corresponding 16.5 mmol/L (75.5%) rise in blood glucose concentration upon induction of diabetes. Despite the three treatments administered, i.e. MET (500 mg of metformin) and VA1 (200 mg of extract) and VA2 (400 mg of extract) the gradual reduction in body weight was seen to persist over the treatment period in tandem with the diabetic control, but at varying rates (Figure 1a). However, the VA1 treated rats showed a comparative advantage in weight control over the animals in VA2 or MET groups. On the other hand a noticeable stepwise reduction in blood glucose was observed in all treatment groups until the end of the study (Figure 1b). The glucose-lowering action was more profound and statistically significant on days 11 and 14: 41.65% and 48.15% for MET; 29.18% and 38.08% for VA1 and 14.79% and 19.61% for VA2 respectively. It appears from this observation that body weight control could be an important index in progress evaluation of any glucose-lowering intervention programme in diabetes therapy.

### Glycolytic target genes GK, HK and PFK

**Figure 2:**
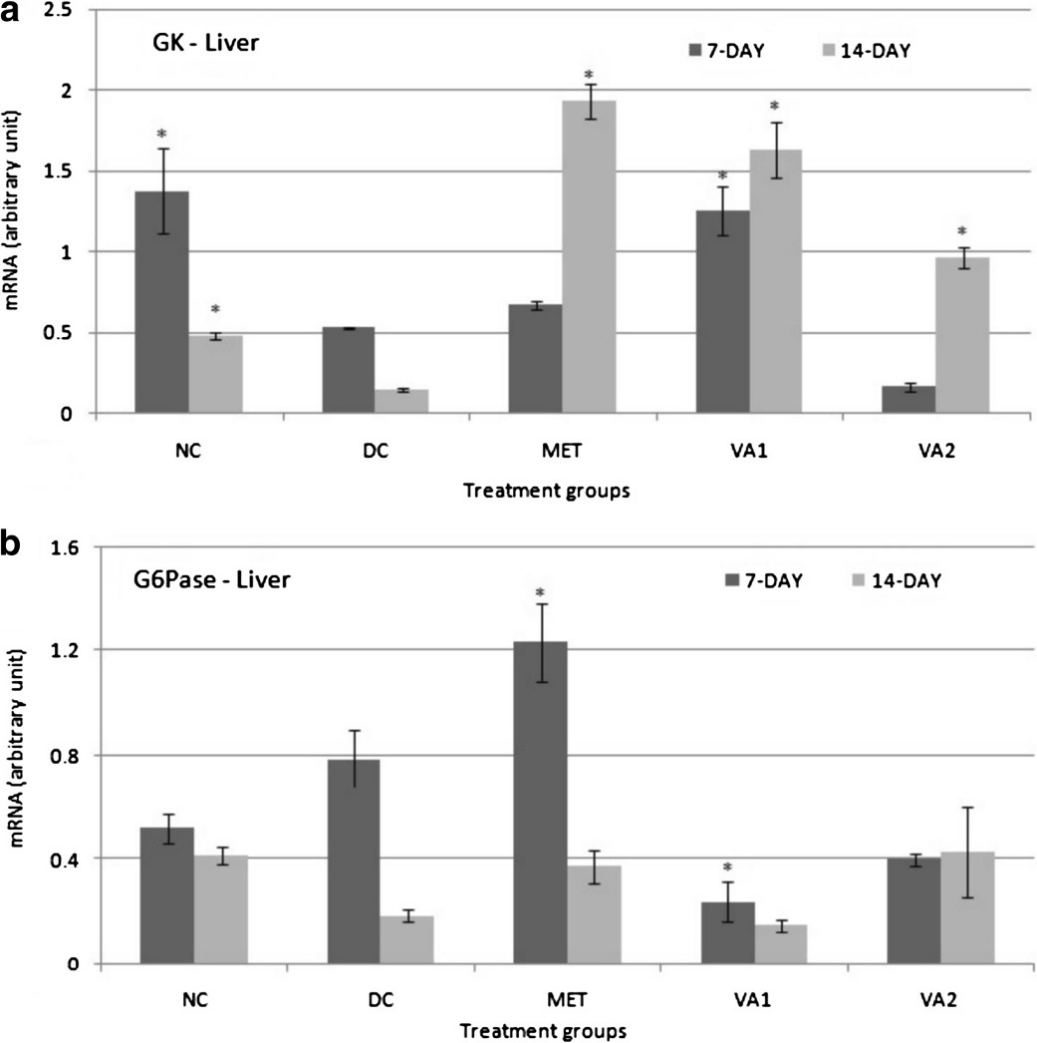
**Glucokinase (GK) and glucose 6-phosphatase (G6Pase) expressions in the liver tissues of streptozotocin-induced diabetic rats treated with chloroform fraction of** ***Vernonia amydalina*** **leaves.** Values are expressed as the mean ± SEM; n = 3; * = *P* <0.05 vs DC.

**Figure 3:**
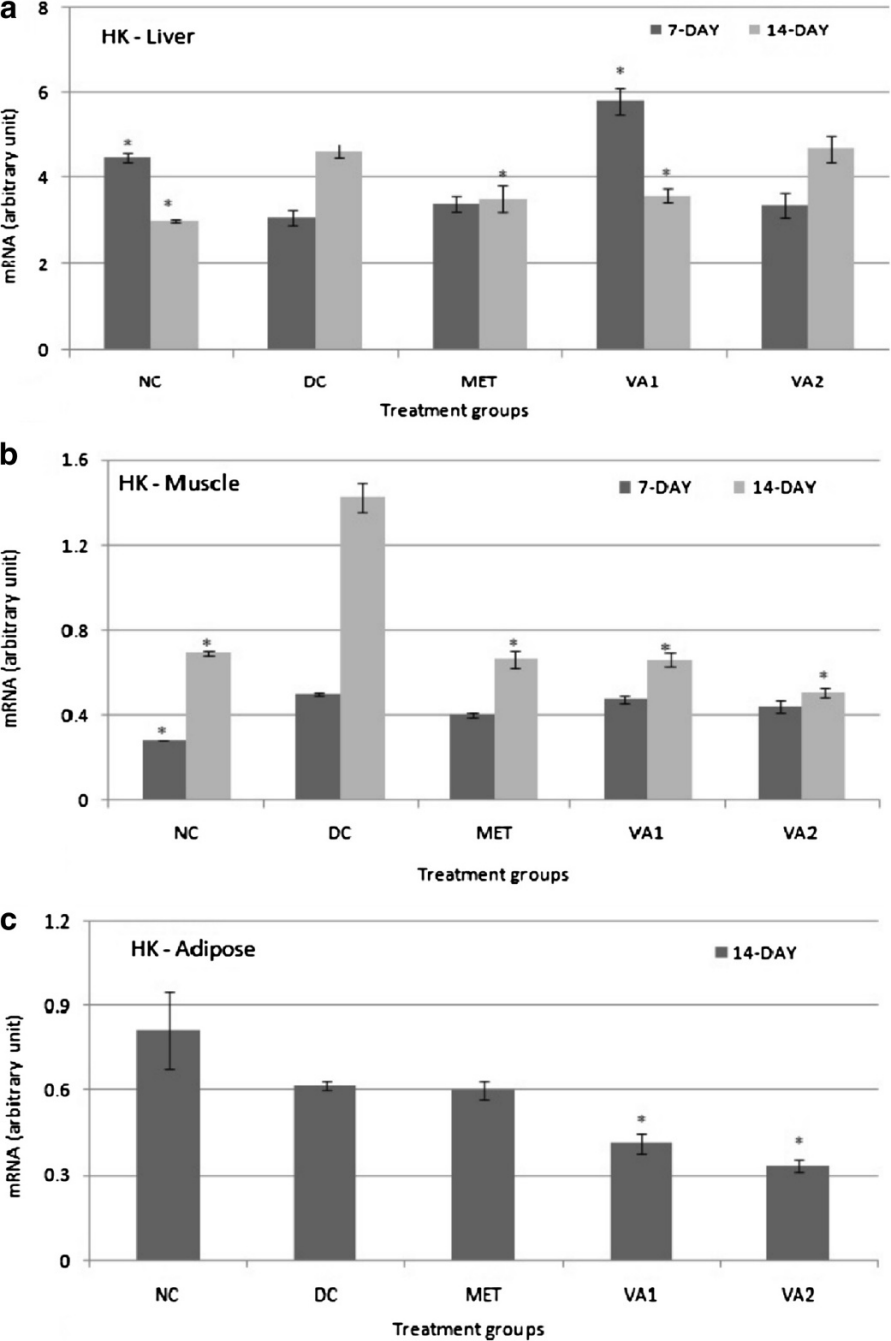
**Hexokinase (HK) expression in the liver (a), muscle (b) and adipose (c) tissues of streptozotocin-induced diabetic rats treated with chloroform fraction of** ***Vernonia amydalina*** **leaves.** Values are expressed as the mean ± SEM; n = 3; * = *P* <0.05 vs DC.

**Figure 4:**
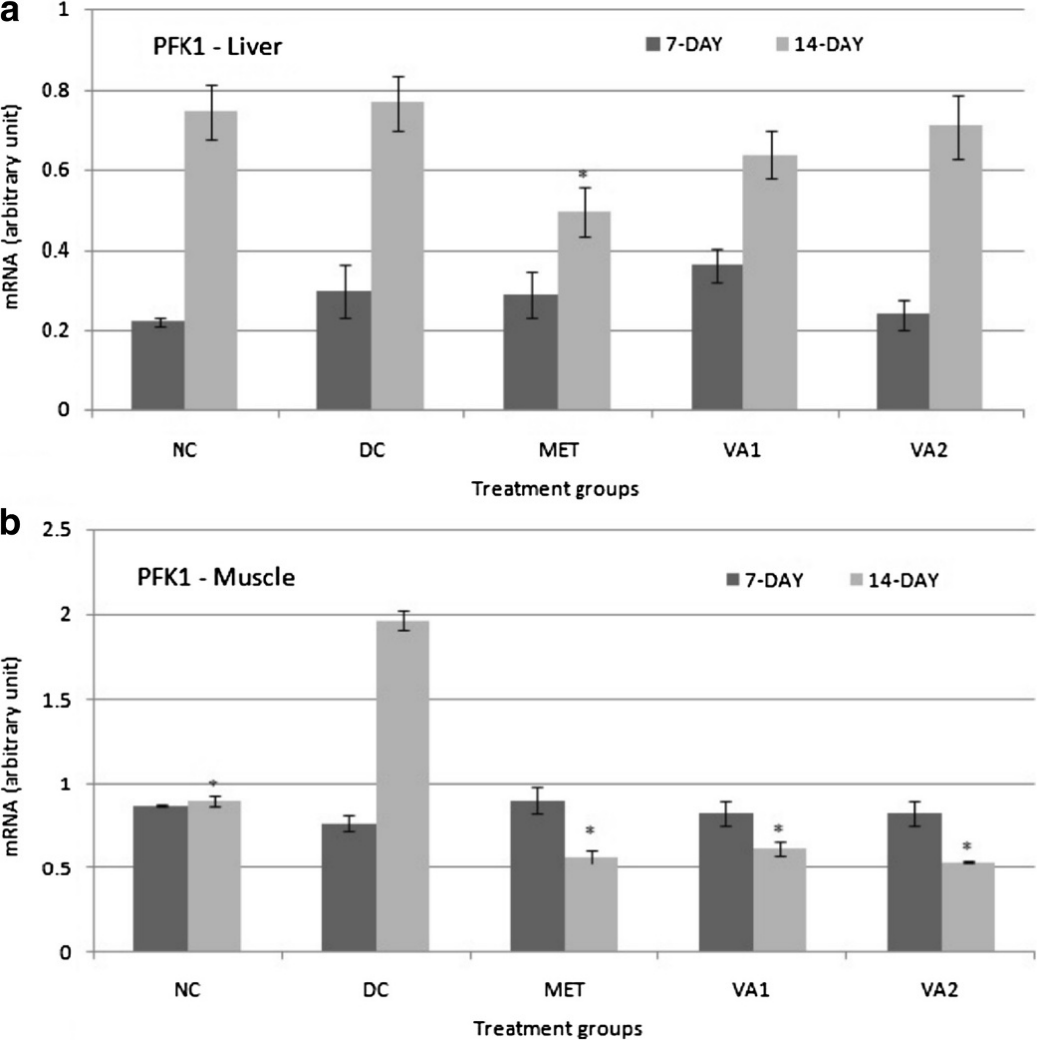
**Phosphofructokinase (PFK) expression in the liver (a) and muscle (b) tissues of streptozotocin-induced diabetic rats treated with chloroform fraction of** ***Vernonia amydalina*** **leaves.** Values are expressed as the mean ± SEM; n = 3; * = *P* <0.05 vs DC.

mRNA levels of three glycolysis - controlled genes namely glucokinase (GK), hexokinase (HK) and phosphofructokinase (PFK) were determined in liver, muscle and adipose tissues of the STZ-induced diabetic rats by conventional PCR, with glyceraldehydes 3 –phosphate (GADPH) as a reference gene (Figures 2, 3 and 4). Following intervention with the chloroform extract and the standard metformin for 7 and 14 days, significant changes were observed in the level of the measured genes. Precisely, a 14-day administration of 200 mg and 400 mg of VA extracts and metformin (500 mg) caused a significant increase (*P* <0.05) in GK gene expression by 8.0, 4.5 and 9.5 folds in the liver tissue of STZ-induced diabetic rats respectively (Figure 2a). On agarose gel electrophoresis stained with ethedium bromide, the GK gene was not expressed in the muscle and adipose tissues. Converse to the impact on GK geneexpression, the HK gene was down-regulated by 1.29 and 1.25 fold (*P* <0.05) in the liver tissues of MET and VA1 treated animals respectively (Figure 3a). A down-regulation in the expression of the HK gene was also observed in muscle tissue - 2.15, 2.15 and 2.80 folds (*P* <0.05) in the MET, VA1 and VA2 groups respectively (Figure 3b). In the adipose tissue, only the VA extract could effectively down-regulate the HK gene expression by 1.49 and 1.84 folds for VA1 and VA2 groups (*P* <0.05) respectively (Figure 3c). Metformin administration exerted no significant changes in the HK gene of the adipose tissue. The densitometry scanning data indicated no significant effect of extract treatments on the liver PFK-1 gene (Figure 4a), but a significant down-regulated expression (*P* <0.05) in the muscle tissue – 3.17, 2.12 and 3.45 folds in the rats in MET, VA1, and VA2 groups respectively (Figure 4b).

### Gluconeogenic target genes – G6Pase, F16BP and PEPCK

**Figure 5:**
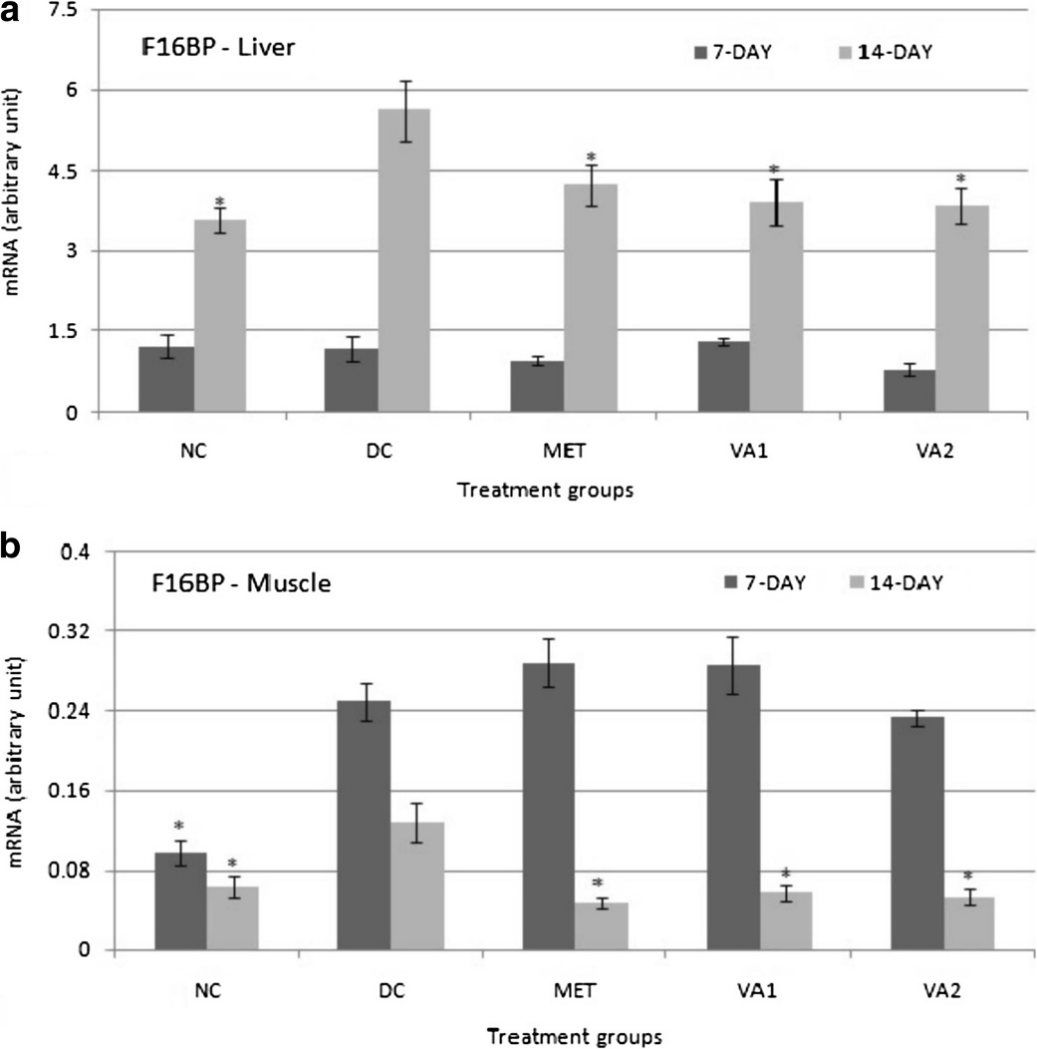
**Fructose 1, 6-bisphosphatase (F16BP) expression in the liver (a) and muscle (b) tissues of streptozotocin-induced diabetic rats treated with chloroform fraction of** ***Vernonia amydalina*** **leaves.** Values are expressed as the mean ± SEM; n = 3; * = *P* <0.05 vs DC.

**Figure 6:**
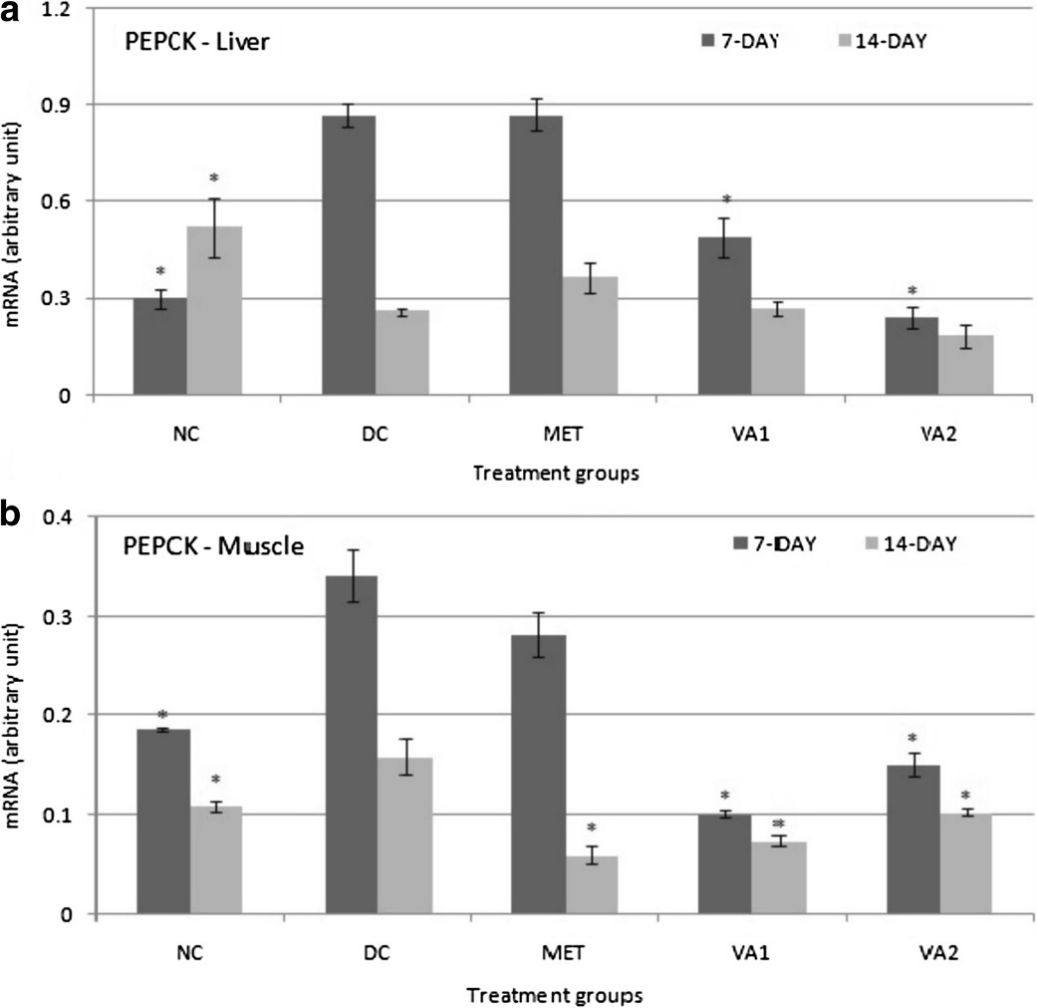
**Phosphoenol pyruvate carboxykinase (PEPCK) expression in the liver (a) and muscle (b) tissues of streptozotocin-induced diabetic rats treated with chloroform fraction of** ***Vernonia amydalina*** **leaves.** Values are expressed as the mean ± SEM; n = 3; * = *P* <0.05 vs DC.

The expression of three gluconeognic target genes namely glucose 6-phosphatase (G6Pase), fructose 1, 6-bisphosphatase (F16BP) and phosphoenol pyruvate carboxykinase (PEPCK) at the mRNA level was determined in the liver and muscle tissues in order to determine the possible effect of the treatments on glucose modulation via gluconeogenesis. The exclusively hepatocyte-domiciled G6Pase gene was only mildly impacted by the interventions of this study: 3.28 (*P* <0.05) and 1.97 folds decreases in VA1 and VA2 respectively after a 7-day treatment (Figure 2b). The effects of a 14-day treatment were not statistically different from those observed after 7 days. In the liver, whereas a 7-day treatment did not cause any significant change in the F16BP gene expression, after a 14 administration, there was significant 1.32, 1.44 and 1.46 folds down-regulation (*P* <0.05) in the MET, VA1 and VA2 groups respectively (Figure 5a). Similar increases in F16BP gene were also indicated in the muscle tissue after a 14-day treatment (Figure 5b) thus: 2.70, 2.23 and 2.40 folds increases in MET, VA1 and VA2 animals respectively (*P* <0.05). Diabetes induction up-regulated the PEPCK gene expression by 2.88 and 1.83 folds in the liver and muscle tissues respectively, but suppressed it by 3.08 in the adipose relative to the non- diabetic rats (*P* <0.05). After a 7-day intervention with 200 and 400 mg of the VA extracts, the PEPCK gene expression was significantly reduced by 1.78 and 3.60 folds (*P* <0.05) in the liver cells respectively (Figure 6a). The 14- day intervention changes were not as profound in the hepatic cells. In the muscle tissue, the 7-day and 14-day administration of the extract down- regulated the PEPCK gene by 3.38 and 2.15 folds in the VA1 respectively and 2.27 and 1.54 folds in the VA2 respectively (*P* <0.05) (Figure 6b). Similarly, metformin treatment restrained gluconeogenesis by decreasing PEPCK gene expression in a time-dependent manner - 1.21 and 2.65 folds decrease after 7 and 14 days respectively (*P* <0.05).

### Pentose phosphate pathway target gene – G6PDH

**Figure 7:**
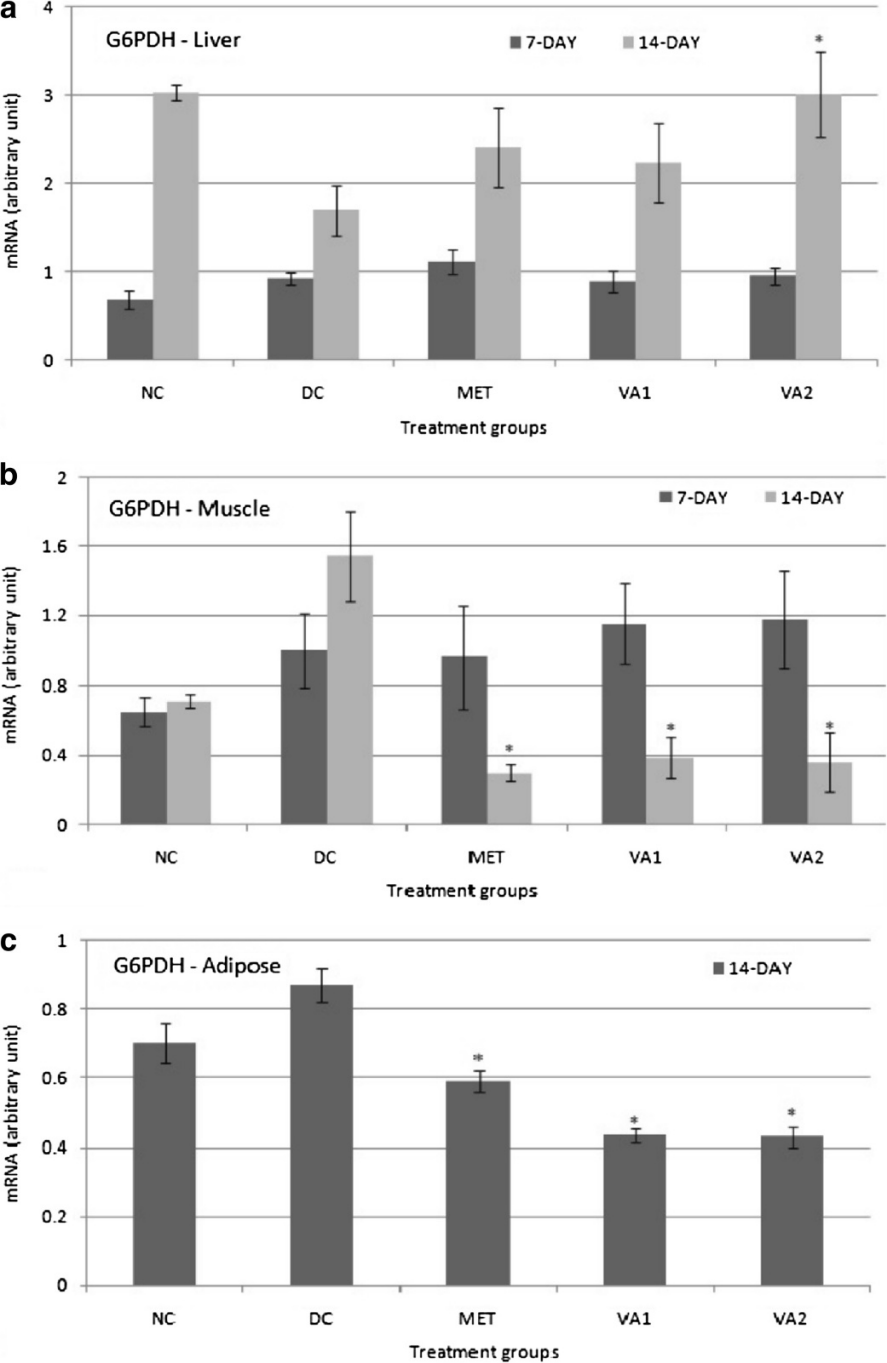
**Glucose 6-phosphate dehydrogenase (G6PDH) expression in the liver (a) muscle (b) and adipose (c) tissues of streptozotocin-induced diabetic rats treated with chloroform fraction of** ***Vernonia amydalina*** **leaves.** Values are expressed as the mean ± SEM; n = 3; * = *P* <0.05 vs DC.

mRNA level of the glucose 6-phosphate dehydrogenase (G6PDH) gene was determined in the liver, muscles and adipose tissues of the study animals in order to assess the impact of VA on glucose oxidation in the pentose phosphate pathway (PPP), a possible glucose modulation mechanism in diabetes mellitus. From the results shown in Figure 7 diabetes induction suppressed liver G6PDH expression by 1.79 folds; however, a 14-day intervention with MET, VA1 and VA2, up-regulated its expression by 1.42, 1.32 and 1.98 folds respectively, implying enhanced glucose utilization in PPP (Figure 7a). A reciprocal of these observed changes were indicated in the muscle and adipose tissues; where diabetes induction alone caused 2.16 and 1.24 folds elevation in the G6PDH gene expression respectively. The administration of MET, VA1 and VA2, significantly down-regulated the expression (*P* <0.05) by 5.14, 3.99 and 4.28 folds in the muscle respectively (Figure 7b); and correspondingly 1.47, 2.00 and 2.03 folds decreases were recorded in the adipose tissue (Figure 7c). By this observation, the extract and metformin indicate a clear tendency towards inhibition of PPP in the insulin-dependent tissues, i.e. the muscle and adipose.

### Cell proliferation and differentiation of target gene - P13K

**Figure 8:**
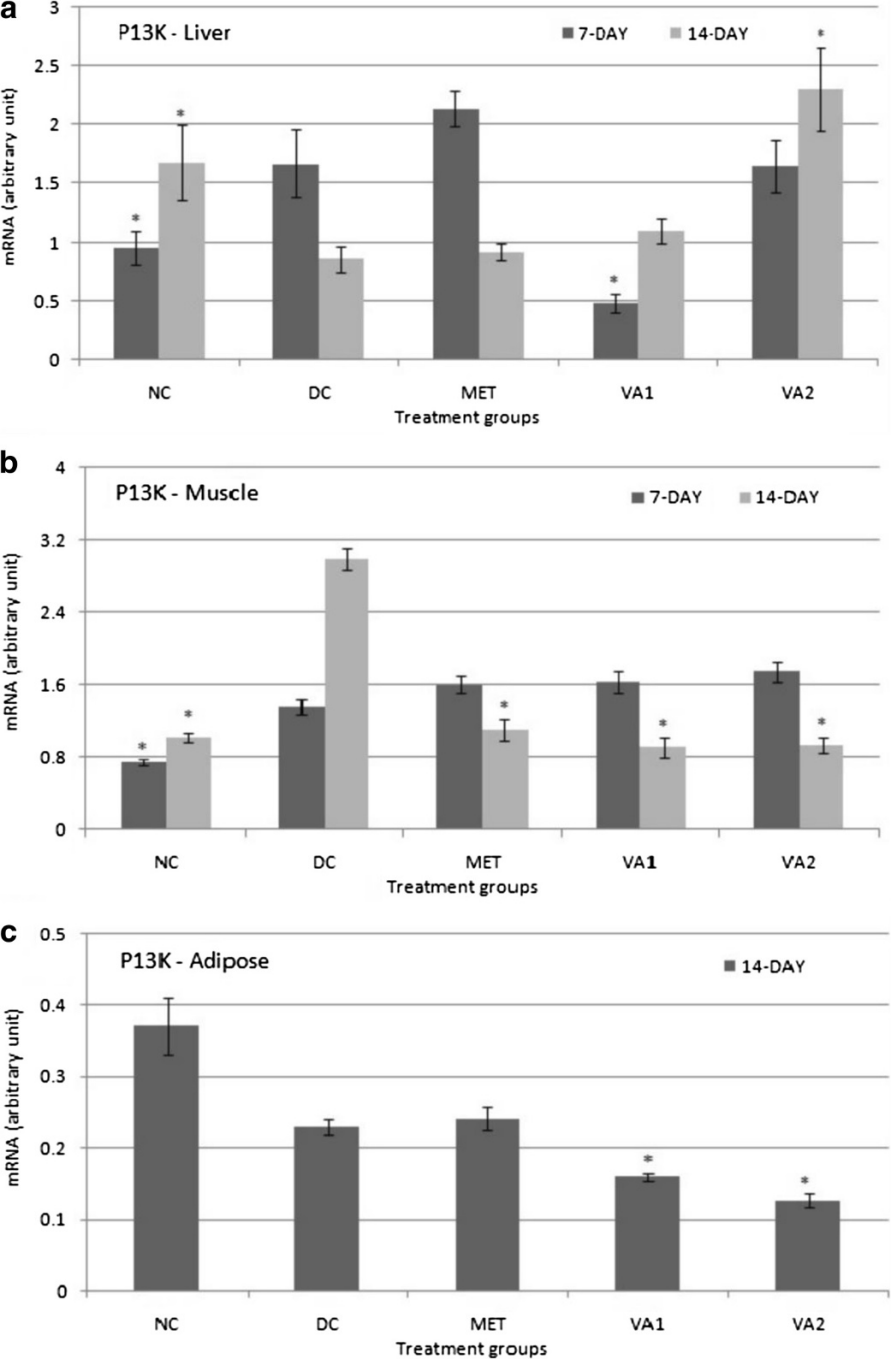
**Phosphatidylinositol 1, 3-kinase (P13K) expression in the liver (a) muscle (b) and adipose (c) tissues of streptozotocin-induced diabetic rats treated with chloroform fraction of** ***Vernonia amydalina*** **leaves.** Values are expressed as the mean ± SEM; n = 3; * = *P* <0.05 vs DC.

The result of P13K gene expression in liver (at the mRNA level) shown in Figure 8 indicates a decrease in its expression in the diabetic control relative to the normal control (*P* <0.05), an indication that differentiation or growth of the liver tissues was down-regulated in diabetic condition. A 14-day intervention with 200 and 400 mg of VA extract exerted a dose-dependent increase in expression - 1.28 and 2.68 folds respectively (Figure 8a). In the muscle, the hitherto increased P13K gene in the diabetic animals (*P* <0.05) decreased by 2.70, 3.28 and 3.21 folds following a 14-day treatment with MET, VA1 and VA2 respectively (*P* <0.05) (Figure 8b). Similarly, a decrease of 1.43 and 1.80 folds was observed in the adipose for VA1 and VA2 groups *P* <0.05) (Figure 8c). These observations suggest a down turn of muscle and adipose tissue differentiation in the intervention groups.

## Discussion

The anti-diabetic properties of *V. amygdalina*, a known medicinal vegetable have been reported in previous studies, particularly in Africa and Asia. However, it remains yet to be known with certainty the effective agent responsible for the anti-diabetic action in the plant and the mechanism of anti-diabetic action of this plant (vegetable). In our recent study an anti-diabetic assay of serial extracts prepared from the leaves of the plant indicated with profound observation that the anti-diabetic activity resides in the non-polar chloroform extract [11]. In furtherance of that study, the present investigation therefore evaluated the effect of the active chloroform extract on key enzymes and proteins of carbohydrate and lipid metabolisms using a molecular approach, with the aim to understand, at least in part, the anti-diabetic mechanism of this medicinal vegetable. Streptozotocin-induced diabetic rat models were used in the 14-day intervention study.

The chloroform extract administered at two dose levels initiated and sustained a step-wise and gradual glucose-lowering action with peak reduction on days 11 and 14. This observation is similar to that in our previous report, where the chloroform extract exerted the highest lowering action on glucose after a 14-day administration [11]. One striking observation in this study that contrasts with previous report on the anti-diabetic activity of VA is the fact that the gradual body weight reduction persisted over the 14-day period, especially in the group that received the higher dose of extract (400 mg). It is likely that fractionation of the whole extract tends to heighten the toxicity effect of VA. Igile and co-workers [14] had shown that mice fed saponin fraction from this plant exhibited features of toxicity including weight reduction at the end of that study. Later on, Ojiako and co-workers [15] reported another study in which extracts from VA leaves were implicated in exerting toxicity action on the study subjects, mice. The impact of the extract on body weight tends to suggest that whole extract from VA leaves might be safer as a medicine compared to its fractions. Tiwari and Rao [16] have in their review indicated and advocated the use of whole plant extracts in preference to isolated and pure compounds from plants articulating several draw backs of pure compounds, prominent amongst which is increased toxicity.

The impact of VA extract on the transcription of GK, HK and PFK-1 genes in the liver, skeletal muscle and adipose was evaluated in order to understand the effect of VA on glycolysis as a glucose modulatory target in diabetes. Whereas the expression of GK, highly decreased in hepatocytes of untreated diabetic rat, was increased at the end of treatment; the HK and PFK-1 genes (decreased or unaltered in diabetic condition, respectively) were decreased significantly at the end of study in the liver, muscle and adipose tissues. These observations, particularly the suppression of the glycolysis pacesetter enzyme gene, PFK-1, clearly indicate that the extract exerted very little or zero impact on glycolysis in respect of its mechanism of anti-diabetic action. The increased expression of hepatic GK obviously suggests glucose activation feed-in into a glucose utilization pathway different from glycolysis, most likely the gluconate pathway. As a confirmation in this study, the G6PDH gene expression which was significantly decreased in untreated diabetic rats, in conformity with previous report [17] became highly expressed at the end of the extract intervention. The G6PDH gene or enzyme is a reported glucose modulatory target of several anti-diabetic agents and of some medicinal plants [17, 18]. Glucose oxidation via the G6PDH pathway primarily produces or generates reducing power –NADPH- needed in synthetic (anabolic) reaction and deactivation of reactive oxygen species (ROS) in the cell (antioxidant action).

It is plausible that the NADPH generated from this activated glucose pathway is used to enhance tissue storage of glucose in form of triglycerides, a process that requires NADPH and for the reductive removal of ROS in a glutathione-dependent pathway. We had shown in a previous study that VA is able to heighten the activity of antioxidant enzymes in diabetic rat models [10]. The gluconate pathway potentiation may also form one of the mechanisms by which VA exerts antioxidant activity thereby ameliorating diabetes mellitus. Insulin production was earlier hypothesized as a valid effect of VA administration in diabetic condition [13] and insulin is known to favour tissue storage of the fuel molecules including TGs. The increased NADPH via G6PDH expression may serve as a boost to the tissue storage process, with the likely aim to clear free fatty acids from circulation.

As emerging from this study, it was also observed that the G6PDH expression decreased in the skeletal muscle and adipose. This could have been a deliberate regulatory mechanism by the tissue to sustain the clearance of ROS, engendered by the hepatocyte generated NADPH, since excess NADPH at any particular time is known to reversibly inhibit the G6PDH enzyme [19]. Moreover, G6PDH activity has been poorly demonstrated in the muscle tissues, unlike the red blood cells, where the G6PDH activity is profound. In a holistic sense, it can be submitted that the VA-induced suppression in hepatic glycolysis was necessary to spare glucose for the pentose phosphate pathway leading to the formation of NADPH needed for lipid synthetic reactions and ROS clearing as well as production of ribose for hepatic cell proliferation.

Phosphatidylinositol 1, 3-kinase, an enzyme involved in regulation of tissue or cell proliferation via production of second messengers for signal transduction, was in this study highly expressed in the hepatic cells, but not in the muscle or adipose. This observation tends to suggests that the hepatocytes, more than the muscle and adipose tissues are more responsible or impacted by the VA extract, hence may be more involved in glucose modulation during VA treatment. The potentiated G6PDH pathway may be responsible for this selective tissue proliferation. The G6PDH pathway is known to generate ribose 5-phosphate in the non-oxidative phase, which can be utilize in the biosynthesis of nucleotides required for the proliferation of cells. Additionally, the NAPDH produced by the same pathway is also an indispensable ingredient needed for deoxyribose nucleotide synthesis [19]. Deoxyribose nucleotides and not ribose nucleotides are needed for cell proliferation. With respect to the hepatocyte-centered action, VA tends to behave in a manner similar to metformin, a typical biguanide. VA extract was shown to act in a manner similar to metformin in our previous studies [11]. It has been noted earlier that biguanides, such as metformin inhibit hepatic gluconeogenesis to achieve anti-diabetic effect [6]. The inhibition of gluconeogenesis by VA extract administration was also clearly demonstrated in the present study as heightened expression of the G6Pase, F16BP and PEPCK genes, the key regulatory enzymes of gluconeogenesis. This observation puts VA leaves in the same category with known plants and compounds such as *Eugenea jambolana*[20], rutin [3] and vernadate [17] with demonstrated hepatic gluconeogenesis inhibition as their anti-diabetic mechanism, besides biguanides which are standard anti-diabetic drugs.

## Conclusions

Besides contributing to the scientific validation of the anti-diabetic properties of *Vernonia amygdalina* growing in Malaysia, data emerging from this study on the effect of the plant extract on the expression of selected genes clearly suggest that *Vernonia amygdalina* may exert anti-diabetic or glucose-lowering action by a simultaneous suppression of gluconeogenesis and potentiation of glucose oxidation via the pentose phosphate pathway almost exclusively in the liver.
